# The role of glycogen synthase kinase-3β (GSK-3β) in endometrial carcinoma: A carcinogenesis, progression, prognosis, and target therapy marker

**DOI:** 10.18632/oncotarget.8485

**Published:** 2016-03-30

**Authors:** Shuo Chen, Kai-Xuan Sun, Bo-Liang Liu, Zhi-Hong Zong, Yang Zhao

**Affiliations:** ^1^ Department of Gynecology, The First Affiliated Hospital of China Medical University, Shenyang 110001, China; ^2^ Department of Biochemistry and Molecular Biology, College of Basic Medicine, China Medical University, Shenyang 100013, China

**Keywords:** endometrial carcinoma, GSK-3β, microRNA-129, AZD 1080, tumorigenesis and progression

## Abstract

**Purpose:**

Glycogen synthase kinase-3β (GSK-3β) is a serine/threonine kinase involved in cancer development. Herein, we demonstrated the role of GSK-3β in endometrial cancer (EC) and identified new therapeutic targets.

**Results:**

GSK-3β was overexpressed in EC tissues, and was positively correlated with International Federation of Gynecology and Obstetrics (FIGO) staging, dedifferentiation, and myometrial infiltration depth. Besides, GSK-3β overexpression predicted lower cumulative and relapse-free survival rate. si-GSK-3β transfection suppressed cell proliferation, migration, invasion, and promoted cell apoptosis through downregulating NF-kB, Cyclin D1 and MMP9 expression whereas upregulating P21 expression. Bioinformatic predictions and dual-luciferase reporter assays showed that GSK-3β was a possible target of miR-129. MiR-129 transfection reduced GSK-3β expression, and exhibited the same trend as si-GSK-3β transfection in cell function experiments. The nude mouse xenograft assay showed that miR-129 overexpression may suppress tumor growth through downregulating GSK-3β expression. Further studies showed that AZD1080, a GSK-3β inhibitor, could also inhibit EC cell proliferation, migration and invasion, while induced cell apoptosis through modulating relevant genes downstream of GSK-3β signaling.

**Experimental Design:**

GSK-3β expression was determined in EC tissue and normal endometrial tissues by immunohistochemistry. After GSK-3β down-regulation by si-GSK-3β, microRNA-129 mimic transfection or GSK-3β inhibitor exposure, EC cell phenotypes and related molecules were examined.

**Conclusions:**

Our results demonstrate for the first time that GSK-3β may be a novel and important therapeutic target for the treatment of endometrial carcinoma. GSK-3β inhibitor AZD1080 may be an effective drug for treating endometrial carcinoma.

## INTRODUCTION

Endometrial cancer (EC) is one of the most common gynecological malignancies, and its incidence has increased remarkably [1–2]. The 5-year survival is approximately 25–45% for stage III and IV cancers [3]. Therefore, it is essential to dissect the underlying molecular mechanisms of tumorigenesis and development of EC for better diagnosis and treatment.

Glycogen synthase kinase-3β (GSK-3β) is a multifunctional kinase, which inhibit glycogen synthesis by inhibiting glycogen synthase through phosphorylation. More than 40 proteins are substrates of GSK-3β, including transcription factors, cell cycle/survival regulators, and oncogenic/proto-oncogenic proteins [4–5], which correlated with various signaling pathways and cellular functions [6]. GSK-3β has been investigated as a therapeutic target for numerous human diseases, including cancer, because of its diverse cellular functions [7]. Qiao et al. reported that GSK-3β was upregulated at both mRNA and protein levels in hepatocellular carcinoma (HCC) specimens, and the overexpression of p-Ser9-GSK-3β was associated with the poor prognosis [8]. Zeng et al. reported that GSK-3β overexpression indicates poor prognosis of non-small cell lung cancer [9]. However, the role of GSK-3β in endometrial carcinoma is still unclear; therefore, we analyzed the potential molecular mechanism of GSK-3β in endometrial carcinoma and explored its function for the diagnosis and therapy of EC.

## RESULTS

### Expression of GSK-3β correlates with pathogenesis and aggressiveness of EC

**Figure 1:**
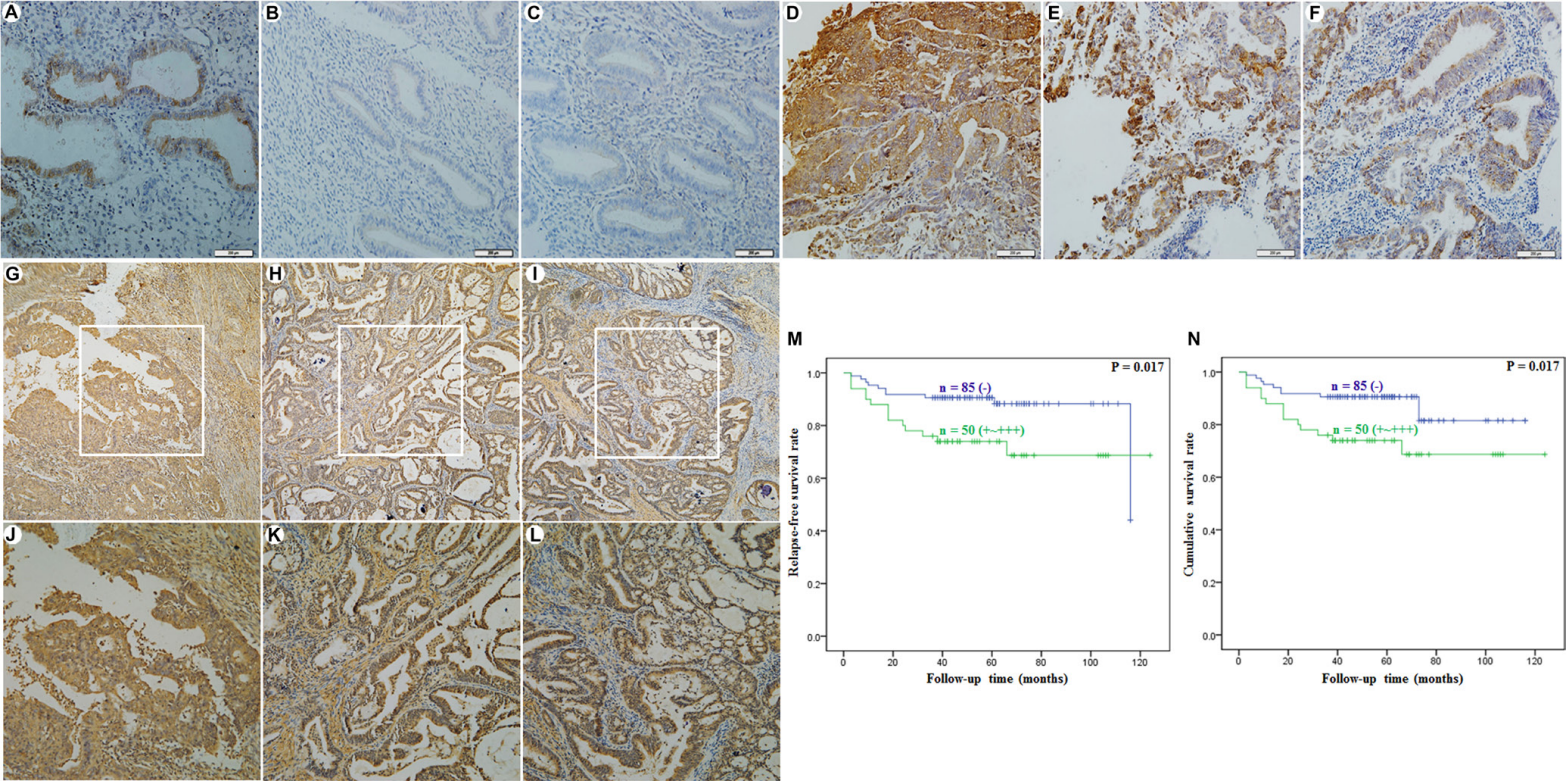
Expression of GSK-3β correlates with pathogenesis and aggressiveness of EC GSK-3β expression levels were stronger in EC tissues (**D**–**I**) compared with normal samples (**A**–**C**). Besides, GSK-3β overexpression was corrected with lower cumulative and relapse-free survival rate (**M** and **N**).

The expression levels of GSK-3β were detected in EC samples and normal samples by Immunohistochemistry. As shown in Figure 1, GSK-3β expression levels were stronger in EC tissues (Figure 1D–1I) compared with normal samples (Figure 1A–1C, details could be found in Table 1). GSK-3β expression was positively correlated with FIGO staging (I & II vs. III & IV, *P* = 0.006), dedifferentiation (Well & Mod vs. Poor, *P* = 0.006), and *the depth of myometrial infiltration* (*< 1/2 vs. ≥* 1/2, *P* = 0.026). Besides, GSK-3β overexpression was corrected with poor cumulative and relapse-free survival rate (Figure 1M and 1N, *P* = 0.017). Details could be found in Table 2.

**Table 1:** GSK-3β expression in endometrial tissues

Groups	GSK-3β (− = 0, + = 1, ++ = 2, +++ = 3)				Total	PR (%)	*X^2^*	*P* value
	0	1	2	3				
**Normal endometrial**	97	48	7	2	154	37.01	4.519	**0.034**
**Endometrial carcinoma**	24	4	0	0	28	14.29		

**Table 2:** Relationship between GSK-3β expression and clinicopathological features of endometrial carcinomas

Variables	GSK-3β (− = 0, + = 1, ++ = 2, +++ = 3)				Total	PR (%)	*X*^2^	*P* value
	0	1	2	3				
**The pathology types**							3.019	0.385
Endometrioid adenocarcinoma	86	46	7	2	141	39.01		
The other pathology types	11	2	0	0	13	15.38		
**Age**							1.131	0.790
< 55	46	24	2	1	73	36.99		
≥ 55	51	24	5	1	81	37.04		
**BMI (WHO)**							2.955	0.450
< 25	57	21	4	1	83	31.33		
≥ 25	40	27	3	1	71	43.66		
**Diabetes**							3.762	0.297
No	69	28	6	1	104	33.65		
Yes	28	20	1	1	50	44.00		
**Hypertension**							1.534	0.700
No	70	34	4	2	110	36.36		
Yes	27	14	3	0	44	38.64		
**FIGO stages**							14.592	**0.006**
I + II	90	34	5	1	130	30.77		
III + IV	7	14	2	1	24	70.83		
**Pathology classification**							22.641	**0.006**
Well + Mod	89	45	7	0	141	36.88		
Poor	8	3	0	2	13	38.46		
**The depth of myometrial infiltration**							8.698	**0.026**
< 1/2	74	30	6	0	110	32.73		
≥ 1/2	23	18	1	2	44	47.73		
**Lymph node metastasis**							7.226	0.065
Negative	92	45	6	1	144	36.11		
Positive	5	3	1	1	10	50.00		
**ER**							3.603	0.299
−	28	12	0	0	40	30.00		
+	69	36	7	2	114	39.47		
**PR**							3.546	0.289
−	23	14	0	0	37	37.84		
+	74	34	7	2	117	36.75		
**Positive ascites cytology**							4.879	0.182
Negative	68	35	5	0	108	37.04		
Positive	29	13	2	2	46	36.96		

Abbreviations: PR = positive rate; Bold represents for P < 0.05.

### si-GSK-3β transfection suppressed EC cell proliferation, increased cell apoptosis and inhibited cell migration and invasion

**Figure 2:**
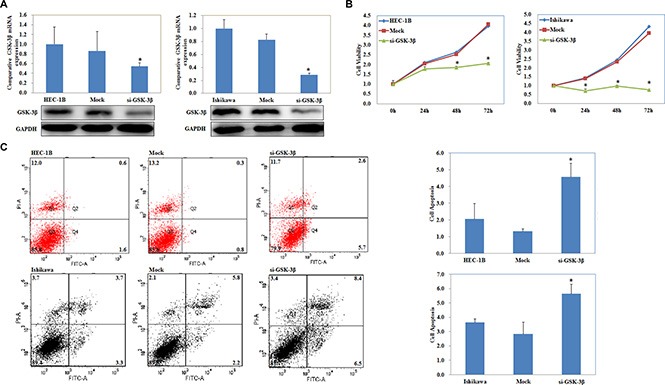
si-GSK-3β transfection suppressed EC cell proliferation, increased cell apoptosis The expression of GSK-3β was significantly downregulated at both the mRNA and protein levels after si-GSK-3β transfection (**A**). MTT assay showed a significant reduction of cell viability after transfection with si-GSK-3β compared with the control group (**B**). si-GSK-3β transfection induced cell apoptosis (**C**) compared with the control group. Results are representative of three separate experiments; data are expressed as the mean ± standard deviation, *P < 0.05.

**Figure 3:**
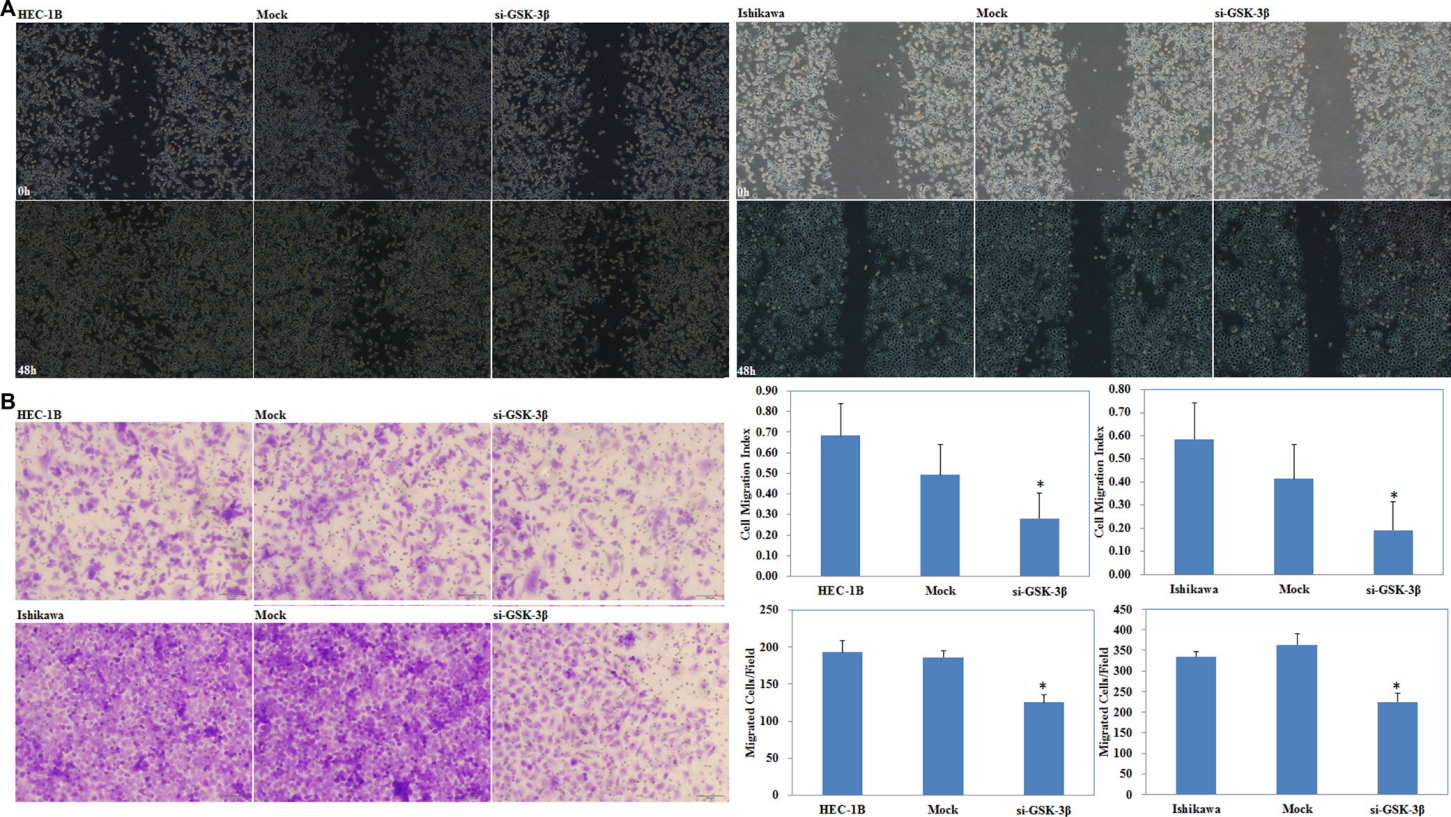
si-GSK-3β transfection inhibited cell migration and invasion si-GSK-3β transfection inhibited cell migration (**A**) and invasion (**B**) ability compared with the control group. Results are representative of three separate experiments; data are expressed as the mean ± standard deviation, *P < 0.05.

si-GSK-3β was transfected into cells, and the expression of GSK-3β was significantly downregulated at both the mRNA and protein levels (Figure 2A). MTT assay showed a significant reduction of cell viability at 48 and 72 h after transfection with si-GSK-3β compared with the control group (*P* < 0.05; Figure 2B). Apoptosis assays demonstrated that cell apoptosis rates were elevated 48 h after transfection with si-GSK-3β compared with control group (*P* < 0.05; Figure 2C). Wound-healing assay showed that cells exhibited a slower closing of the scratch wound after transfection with si-GSK-3β compared with the control group (*P* < 0.05; Figure 3A). Transwell assays showed that cells transfected with si-GSK-3β had a reduced invasive ability compared with the control group (*P* < 0.05; Figure 3B).

### NF-kB, Cyclin D1, MMP9, and P21 expression is regulated by si-GSK-3β transfection

**Figure 4:**
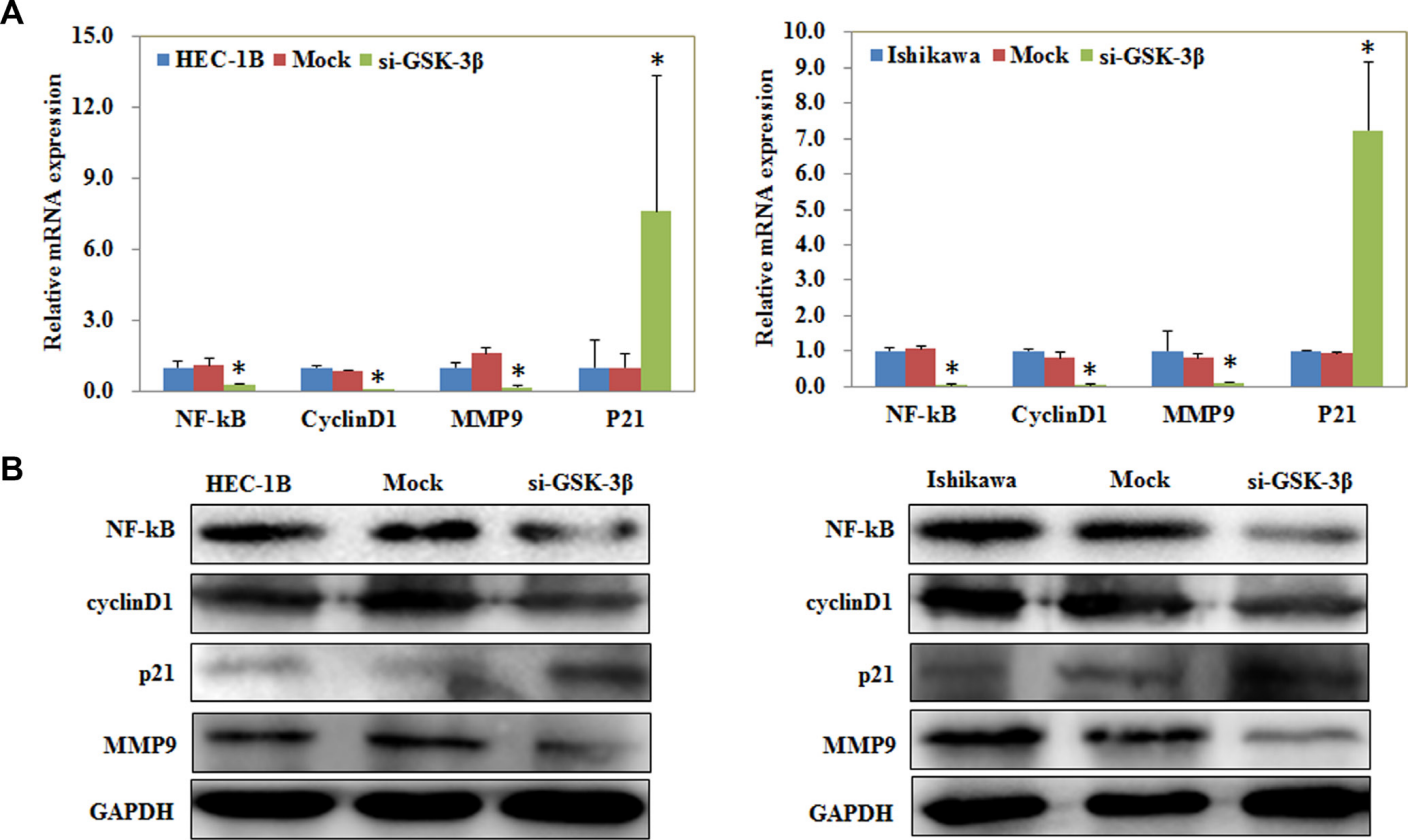
NF-kB, Cyclin D1, MMP9, and P21 expression is regulated by si-GSK-3β transfection Following transfection of si-GSK-3β, qRT-PCR and Western blot analysis showed decreased levels of NF-kB, Cyclin D1 and MMP9 at the mRNA (**A**) and protein (**B**) levels, however, P21 expression increased at the mRNA and protein levels compared with the negative control. *P < 0.05.

Following transfection of si-GSK-3β, qRT-PCR and Western blot analysis showed decreased levels of NF-kB, Cyclin D1 and MMP9 at the mRNA (*P* < 0.05; Figure 4A) and protein (Figure 4B) levels, however, P21 expression increased at the mRNA and protein levels compared with the negative control.

### GSK-3β downregulation by miR-129 overexpression suppressed EC cell proliferation, increased cell apoptosis and inhibited cell migration and invasion

**Figure 5:**
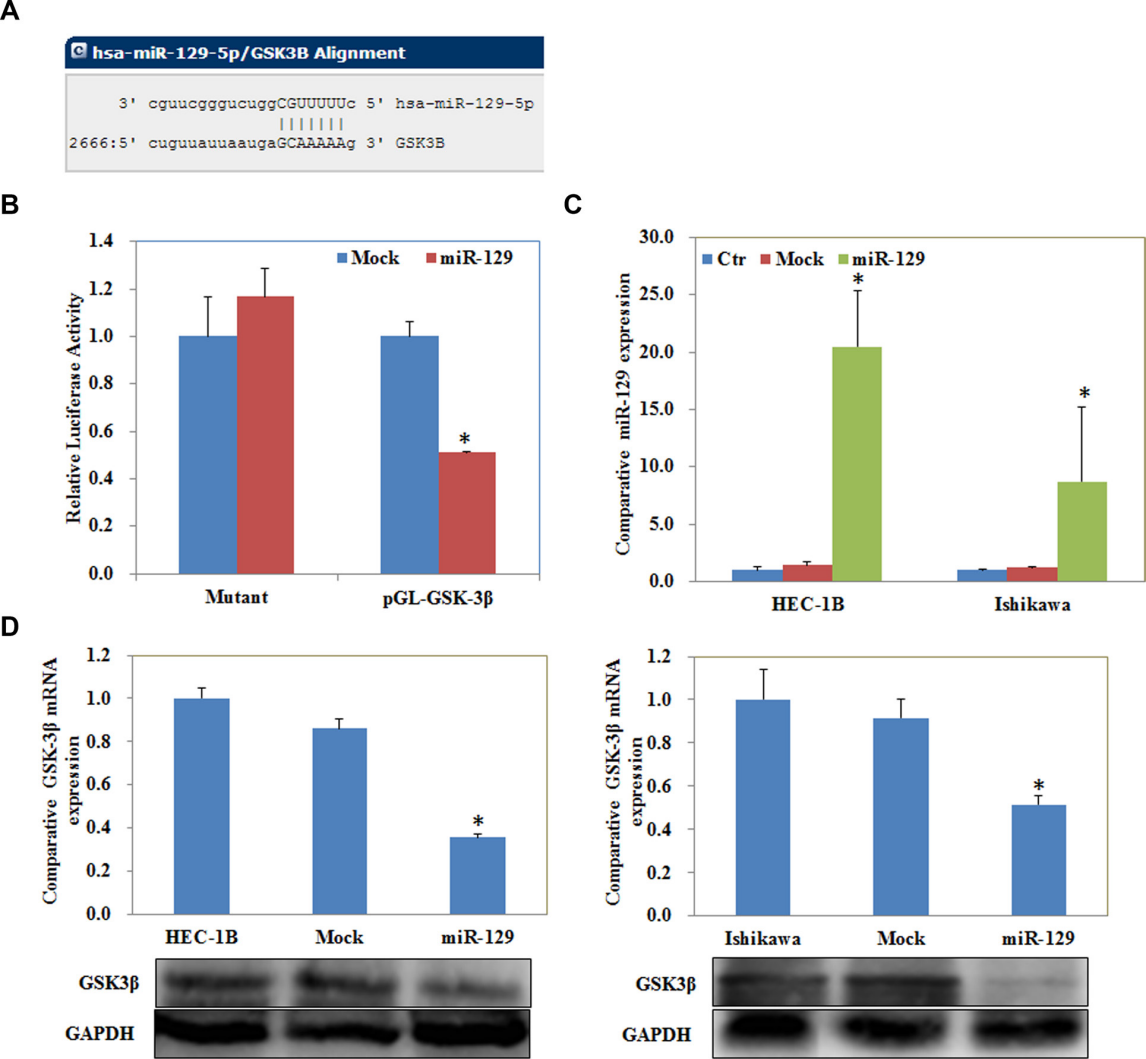
GSK-3β was a target of miR-129 The microRNA.org prediction website showed a miR-129 binding site in the 3′UTR of GSK-3β (**A**). Luciferase reporter assays showed that miR-129 might directly bind to the 3′UTR of GSK-3β (**B**). qRT-PCR and Western blot analysis showed that miR-129 overexpression (**C**) reduced GSK-3β expression at both the mRNA and protein levels (**D**). Results are representative of three separate experiments; data are expressed as the mean ± standard deviation, *P < 0.05.

**Figure 6:**
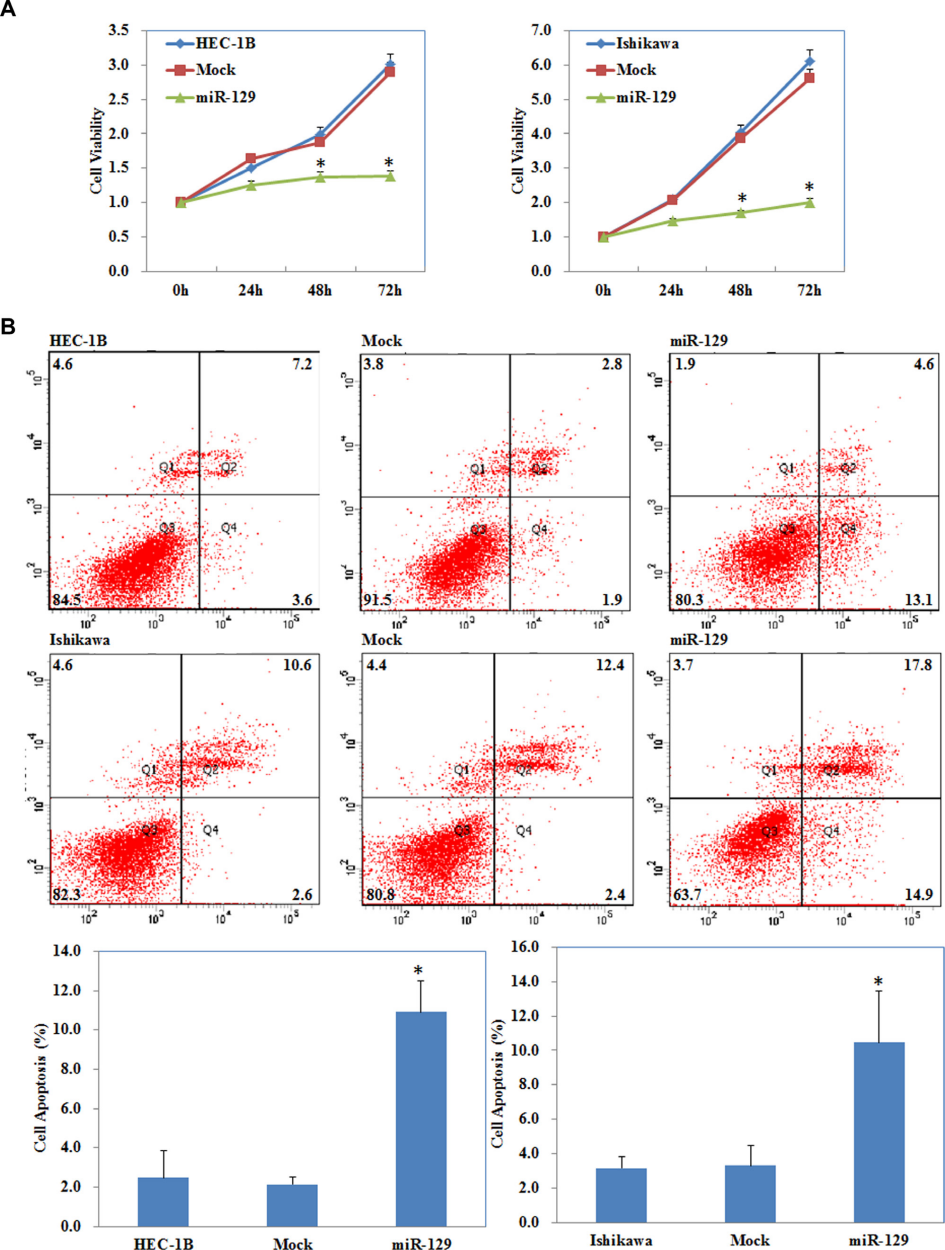
miR-129 overexpression suppressed EC cell proliferation, increased cell apoptosis miR-129 transfection suppressed cell proliferation (**A**), induced cell apoptosis (**B**) compared with the control group. Results are representative of three separate experiments; data are expressed as the mean ± standard deviation, *P < 0.05.

**Figure 7:**
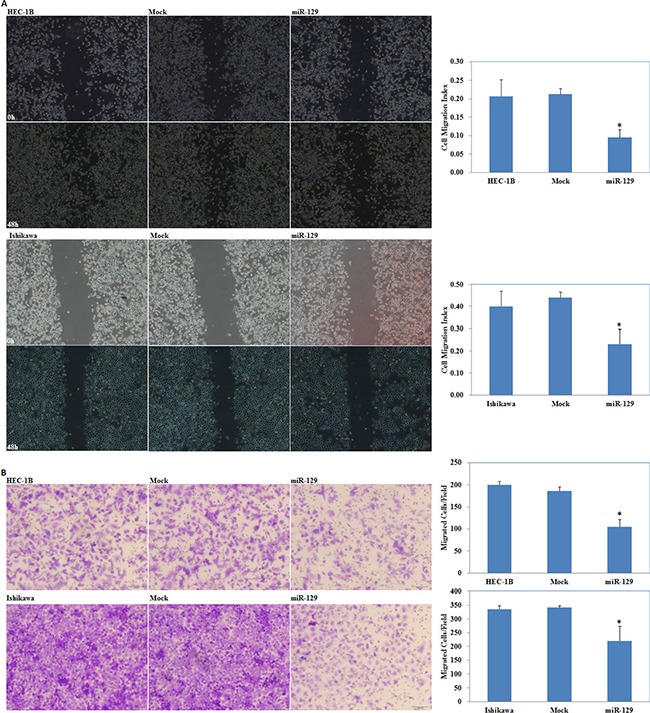
GSK-3β downregulation by miR-129 overexpression inhibited cell migration and invasion miR-129 transfection inhibited cell migration (**A**) and invasion (**B**) ability compared with the control group. Results are representative of three separate experiments; data are expressed as the mean ± standard deviation, *P < 0.05.

We located a miR-129 binding site in the 3′UTR of GSK-3β using the microRNA.org prediction website (Figure 5A). Luciferase reporter assays convinced this prediction (*P* < 0.05; Figure 5B). qRT-PCR and Western blot analysis showed that miR-129 overexpression by miR-129 transfection (*P* < 0.05; Figure 5C) reduced GSK-3β expression at both the mRNA and protein levels (*P* < 0.05; Figure 5D). The MTT assay showed a significant reduction of cell viability at 48 and 72 h after miR-129 mimic transfection compared with the control group (*P* < 0.05; Figure 6A). Apoptosis assays demonstrated that cell apoptosis rates were elevated 48 h after transfection with the miR-129 mimics compared with the control group (*P* < 0.05; Figure 6B). The wound-healing assay showed that following transfection with miR-129 mimics, cells exhibited a slower closing of the scratch wound compared with the control group (*P* < 0.05; Figure 7A). Transwell assays showed that miR-129 mimic transfected cells exhibited a reduction in invasive ability compared with the control group (*P* < 0.05; Figure 7B).

### MiR-129 overexpression regulate NF-kB, Cyclin D1, MMP9, and P21 expression *in vitro*, suppresses the tumorigenicity and development of endometrial carcinoma cells *in vivo*

**Figure 8:**
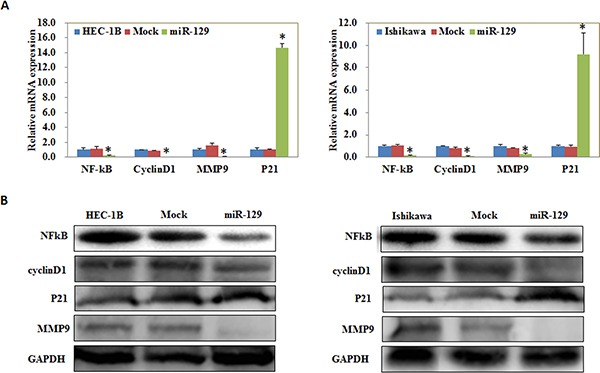
MiR-129 overexpression regulate NF-kB, Cyclin D1, MMP9, and P21 expression *in vitro* qRT-PCR and Western blot demonstrated decreased expression of NF-kB, Cyclin D1 and MMP9 at the mRNA (**A**) and protein (**B**) levels, while P21 expression was increased after transfection of miR-129 mimics compared with the negative control. *P < 0.05.

**Figure 9:**
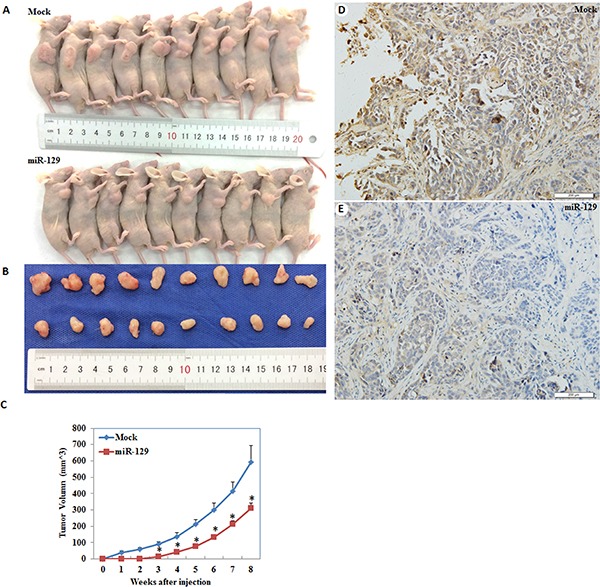
MiR-129 overexpression suppresses the tumorigenicity and development of endometrial carcinoma cells *in vivo* Mice injected with the HSA-129 transfected cells showed slower tumorigenicity (**C**) and smaller volume of tumors during the same observation period (**A**–**B**). Besides, GSK-3β expression was significantly downregulated in the HSA-129 group compared with the control group in nude mice tumor tissues (**D**–**E**). *P < 0.05.

qRT-PCR and Western blot demonstrated decreased expression of NF-kB, Cyclin D1 and MMP9 at the mRNA and protein levels, however, P21 expression was increased after transfection of miR-129 mimics compared with the negative control (Figure 8A and 8B). Nude mice xenograft assays showed that mice injected with the HSA-129 transfected cells showed slower tumorigenicity compared with the control group and exhibited a smaller volume of tumors during the same observation period (Figure 9A–9C). Immunohistochemical analysis demonstrated that GSK-3β expression was significantly downregulated in the HSA-129 group compared with the control group in nude mice tumor tissues (Figure 9D–9E).

### GSK-3β inhibitor AZD1080 suppressed EC cell proliferation, migration and invasion, increased cell apoptosis and modulate NF-kB, Cyclin D1, MMP9, and P21 expression

**Figure 10:**
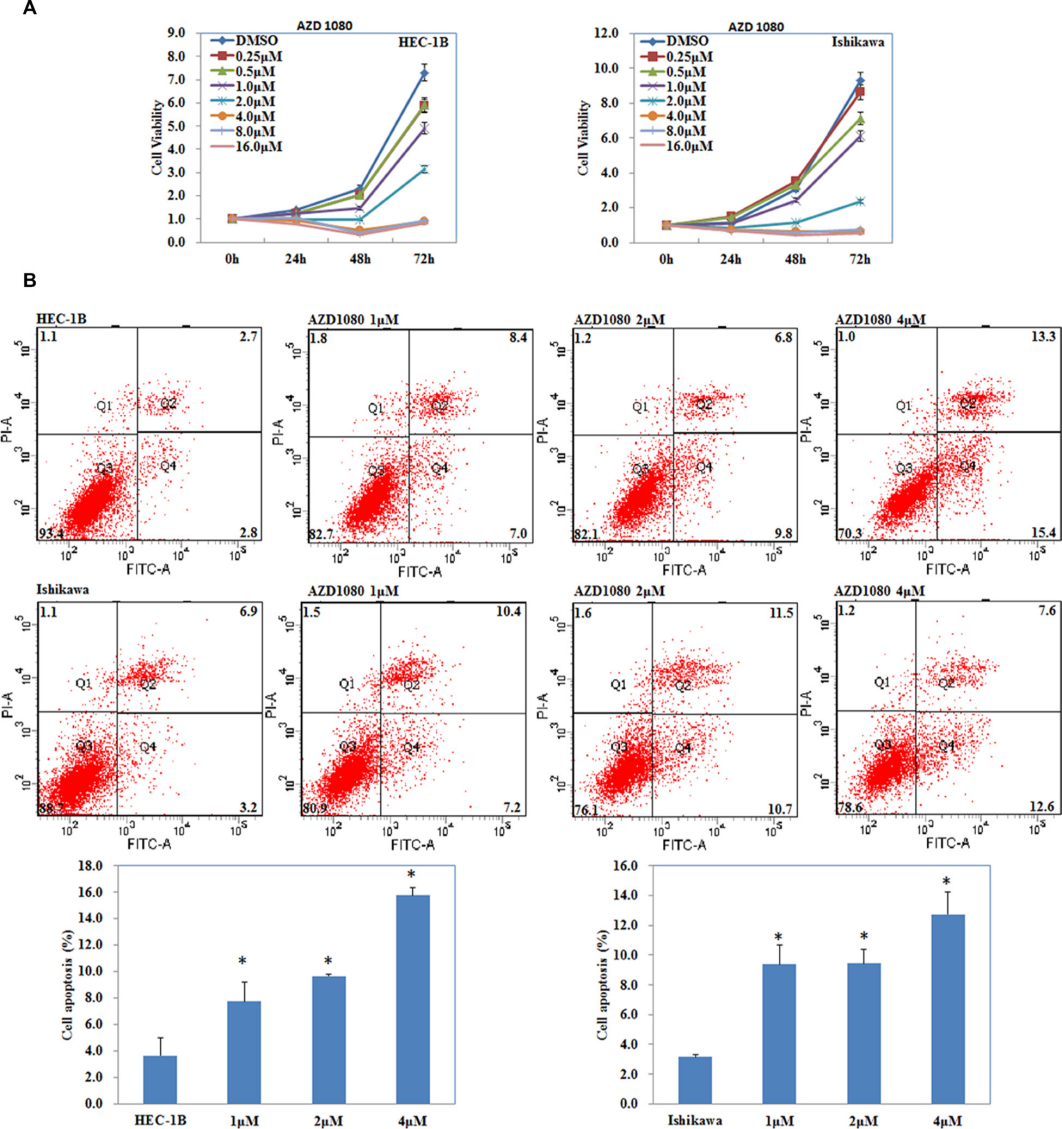
GSK-3β inhibitor AZD1080 suppressed EC cell proliferation, increased cell apoptosis AZD1080 exposure inhibited cell proliferation (**A**), induced cell apoptosis rates (**B**) compared with the control group, and was concentration-dependently. Results are representative of three separate experiments; data are expressed as the mean ± standard deviation, *P < 0.05.

**Figure 11:**
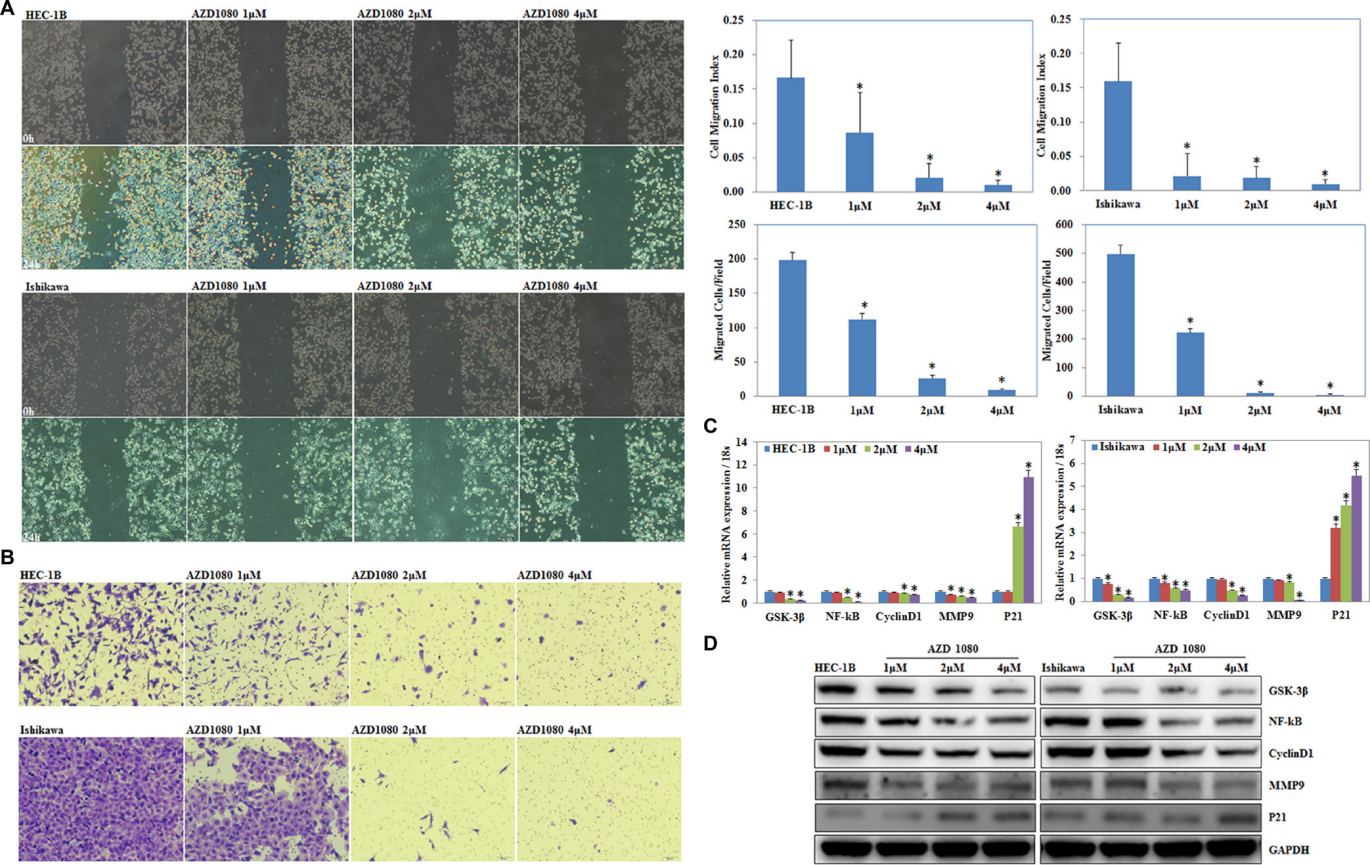
GSK-3β inhibitor AZD1080 suppressed EC cell migration and invasion and modulate NF-kB, Cyclin D1, MMP9, and P21 expression AZD1080 exposure suppressed cell migration (**A**) and invasion ability (**B**) compared with the control group, and was concentration-dependently. AZD1080 exposure also decreased mRNA (**C**) and protein (**D**) expression of NF-kB, Cyclin D1 and MMP9 while increased P21 expression when exposed to AZD1080. Results are representative of three separate experiments; data are expressed as the mean ± standard deviation, *P < 0.05.

The MTT assay showed a significant reduction of cell viability started from 0.5 μM when exposed to AZD1080 compared with the DMSO group, and thus the concentration of 1.0, 2.0, 4.0 μM was used for further study (*P* < 0.05; Figure 10A). AZD1080 induced cell apoptosis rates (*P* < 0.05; Figure 10B), suppressed cell migration (*P* < 0.05; Figure 11A) and invasive ability (*P* < 0.05; Figure 11B) compared with the control group, and was concentration-dependently. RT-PCR (Figure 11C) and Western blot (Figure 11D) results demonstrated decreased expression of NF-kB, Cyclin D1 and MMP9 while increased P21 expression when exposed to AZD1080.

## DISCUSSION

GSK-3β phosphorylates various proteins involved in glycogen synthesis, gene expression, cell migration, cell cycle, cellular architectural pathways, and oncogenesis [10–13]. Our results showed that GSK-3β expression was significantly higher in EC tissues than in normal endometrial tissues. In addition, GSK-3β expression was positively related to histological differentiation, FIGO stage, and the depth of myometrial infiltration, which together demonstrate a poor prognosis. Therefore, we hypothesize that GSK-3β may play an oncogenic role in EC. Thus, we transfected si-RNA GSK-3β constructs to deplete GSK-3β levels in order to observe changes in the occurrence and development of EC cells using a series of functional cell assays. The results demonstrated that depletion of GSK-3β suppressed the proliferation, migration, and invasion of cells, and promoted the apoptosis of EC cells. Taken together, our results demonstrated an inhibition of EC after GSK-3β was depleted. Shi et al. reported that CD151 overexpression upregulated the expression of MMP9 through the PI3K/Akt/GSK-3β/Snail pathway in hepatocellular carcinoma [14]. A previous study showed that the downregulation of cyclin D1 degradation through the constitutive active AKT/GSK-3β pathway inhibited cell proliferation [15]. Deng et al. found that inhibition of GSK-3β suppressed NF-kB activity [16]. Additionally, Yohn et al. reported that GSK-3β inhibition causes an increase in p21 in bladder cells [17]. Therefore, we detected the expression levels of NF-kB, Cyclin D1, MMP9, and P21 after transfection with si-GSK-3β and found that NF-kB, Cyclin D1 and MMP9 expression were downregulated, whereas P21 expression was upregulated. Thus, we hypothesize that GSK-3β can promote the carcinogenesis and progression of EC and that downregulating GSK-3β may hinder the development of EC cells through regulating a series of related downstream genes. Therefore, GSK-3β may be a new therapeutic target of EC.

Next, we investigated the possible upstream regulatory genes of GSK-3β. MiRNAs are a class of small non-coding RNAs that negatively regulate gene expression at the post-transcriptional level [18–26]. MiRNA 129 has been reported to have great potential as a therapeutic agent in select tumors including bladder cancer [27], colorectal cancer [28], gastric cancer [29], and lung cancer [30], and may inhibits cancer cell proliferation, metastasis and invasion through targeting relevant genes such as ETS1 [31], CDK6 [32], PAK5 [33]. Our predicted seed region in the 3′ UTR of GSK-3β revealed that GSK-3β is a target of miR-129, which was also convinced by our luciferase reporter assay results. Therefore, we transfected miR-129 mimics into EC cells and found that the GSK-3β expression both at the mRNA and protein level was downregulated. Further experiments showed that miR-129 overexpression also suppressed the proliferation, migration, invasion of cells, and promoted the apoptosis of EC cells, which were completely in line with our si-GSK-3β experimental results. Next, we detected the mRNA and protein expression levels of NF-kB, Cyclin D1, MMP9, and P21 in EC cells following transfection of miR-129. We found that NF-kB, Cyclin D1 and MMP9 expression was downregulated, whereas, P21 expression was upregulated, likewise, these results were similar to our si-GSK-3β transfected cells. Therefore, we hypothesize that GSK-3β may be downregulated by miR-129 in order to suppress the development of EC. Lastly, we performed nude mice xenograft assays to elucidate if miR-129 could affect the expression level of GSK-3β and further influence the tumorigenicity and development of EC *in vivo*. As predicted, miR-129 overexpression significantly downregulated GSK-3β expression and suppressed tumorigenicity and slowed down the progression of EC *in vivo*. These results demonstrate the oncogenic role of GSK-3β in EC. The downregulation of GSK-3β is an effective way to inhibit EC tumorigenesis and progression, and miR-129 is an effective and important miR.

AZD1080, a novel GSK-3β inhibitor, has been reported to play a pivotal role in attenuating the downstream detrimental effects of signaling pathways activated by multiple stimuli relevant to Alzheimer's disease [34]. Their results indicate for the first time that AZD1080 has the ability to inhibit the GSK-3β enzyme in humans at 1–10 μmol/kg, and could produce a dose-dependent reduction through acute oral treatment. Taken our previous studies in sight, we suggest that AZD1080 may have potential in inhibiting endometrial carcinoma progression. Our studies showed significant reduction of endometrial cancer cell viability from 0.5 μM when exposed to AZD1080. Besides, AZD1080 induced cell apoptosis, suppressed cell migration, and invasion ability, which was concentration-dependently. Besides, AZD1080 exposure decreased NF-kB, Cyclin D1 and MMP9 expression while increased P21 expression, thus we guess that AZD1080 may be an effective drug for treating endometrial carcinoma. As it as a new, potential anti-cancer drug, further *in vitro* tests and clinical studies are needed before it can be used to treat endometrial carcinoma clinically.

The present study is the first to indicate the oncogenic role of GSK-3β. GSK-3β may be a novel therapeutic target for treatment of EC. The inhibition of GSK-3β expression by miR-129 may prove to be an effective therapeutic strategy for EC. GSK-3β inhibitor AZD1080 inhibit endometrial carcinoma cell proliferation, migration and invasion and may be an effective drug for treating endometrial carcinoma.

## MATERIALS AND METHODS

### Endometrial carcinoma specimens

154 endometrial adenocarcinomas and 28 normal endometrial specimens were obtained during surgery at the Department of Gynecology of the First Affiliated Hospital of China Medical University (Shenyang, Liaoning, China). The tumor specimens were independently confirmed by two pathologists. None of the patients had preoperative chemotherapy or radiotherapy. Informed consent was obtained from each patient; all specimens were handled and made anonymous according to ethical and legal standards.

### Cell culture and transfection

The HEC-1B cells were cultured in Modified Eagle's Medium (DMEM; HyClone, Logan, UT, USA). Ishikawa cells were cultured in RPMI 1640 medium (HyClone, Logan, UT, USA). The medium was supplemented with 10% fetal bovine serum (FBS), 100 units/ml penicillin, and 100 units/ml streptomycin. Cultures were incubated at 37°C in a humidified atmosphere with 5% CO_2_. All transfections were carried out using Lipofectamine 2000 according to the manufacturer's instructions. MiR-129-5p mimics: 5′-CUUUUUGCGGUCUGGGCUUGC-3′ and 5′-AAGCCCAGACCGCAAAAAGUU-3′. Small interfering RNA (siRNA) targeting human GSK-3β mRNA (si-GSK-3β): 5′-CACUCAAGAACUGUCAAGUdTdT-3′ and 5′-ACUUGACAGUUCUUGAGUGdTdT-3′.

### MTT assay

The cells were seeded at a density of 3,000 cells/well in 96-well plates. At a given time point (0 h, 24 h, 48 h, and 72 h) after transient transfection, MTT solution (5 mg/ml; Solarbio, Beijing, China) was added to the cells and further incubated for 4 h. The MTT solution was removed, 150 μL/well of DMSO was added, and the absorbance was measured at 490 nm using a microplate spectrophotometer (Bio-Tek Instruments, Winooski, VT, USA).

### Apoptosis assay

Apoptosis was quantified using PI staining and flow cytometry with fluorescein isothiocyanate (FITC)-labeled annexin V (BD Pharmingen, USA) following the manufacturer's protocol. Cells were collected 48 h after transfection, washed twice with cold PBS, resuspended at 1 × 10^6^ cells/mL, and mixed with 100 μL of 1 × buffer, 5 μL annexin V-FITC, and 5 μL PI and incubated for 15 min in the dark; 400 μL 1 × buffer was added to the cells, and the cells were subjected to cytometry flow within 1 h.

### Wound-healing assay

Cells were cultured to 85% confluence before scratched with a 200 μl pipette tip. After scratching, cells were washed with PBS three times and cultured in FBS-free medium. Wounds were observed by a microscope and photographed at 0, 24, and 48 h. The nude areas were measured using the Image J software (National Institutes of Health, Bethesda, MD, USA), and the percentage of wound closure was calculated.

### Invasion assay

Matrigel-coated transwell filters (BD Bioscience, San Jose, CA, USA) were used for the invasion assay. Matrigel was used at a dilution of 1:10. Cells (5 × 10^4^/L) suspended in serum-free medium were layered in the upper compartment of the transwell inserts. The bottom chambers contained medium with 10% fetal bovine serum, which served as the chemoattractant. After incubation for 48 h at 37°C, invaded cells at the bottom of the upper chamber were stained with crystal violet and counted under an Olympus fluorescence microscope (Tokyo, Japan).

### Real-time RT-PCR

The total RNA was used to produce cDNA with avian myeloblastosis virus transcriptase and random primers (Takara, Shiga, Japan) according to the manufacturer's protocol. For quantification, cDNA samples were subjected to real-time PCR using the SYBR Premix Ex Taq™ II kit (Takara, Shiga, Japan). The expression levels of genes were normalized to 18s mRNA. Details of the primer sequences could be found in Supplementary Table 1.

### Western blotting

Cells were harvested and lysed using ice-cold RIPA lysis buffer. All denatured protein samples were separated by 10% SDS-polyacrylamide gel electrophoresis (SDS-PAGE) and transferred onto Hybond membranes (Amersham, Munich, Germany). Following blocking for 2 h in 5% fat-free milk, the membranes were incubated with primary antibodies against GSK-3β, NF-kB, Cyclin D1, MMP9, and P21 (1:500, Proteintech, Proteintech Group, USA). Blots were washed with TBST and then incubated with secondary antibodies (1:5000). Bands were visualized using an enhanced chemiluminescence (ECL) system according to the manufacturer's protocol (Santa Cruz Biotechnology, Santa Cruz, CA, USA). Anti-GAPDH (1:2000) was set as the internal control.

### Immunohistochemistry

Paraffin-embedded tissue sections were prepared for analysis of GSK-3β expression. The samples were deparaffinized and rehydrated, then incubated for 20 min in 3% H_2_O_2_ to quench endogenous peroxidase activity. Next, the sections were heated to retrieve the antigen and incubated with 10% goat serum to block non-specific binding. The samples were probed with an anti-GSK-3β primary antibody (1:50), and then an appropriate secondary antibody. After each treatment, the slides were washed three times with TBST for 5 min. The binding was visualized with 3, 39-diaminobenzidine tetrahydrochloride (DAB).

### *In vivo* nude mouse xenograft assay

BALB/c nude mice at 4 to 6 weeks of age were obtained from Vital River Laboratories (VRL; Beijing, China). The animal study was reviewed and approved by National Institutes of Health Guide for the Care and Use of Laboratory Animals with the approval of the China Medical University Animal Care and Use Committee. As previously reported [35], a total of 1 × 10^7^ HEC-1B cells transfected with mutant or wild-type HSA-129 were resuspended in FBS-free culture medium and injected subcutaneously into the right flanks of the nude mice. Eight weeks after injection, the mice were sacrificed. The tumor volume was measured routinely using direct measurement and calculated using the formula (length × width^2^)/2.

### Dual-luciferase reporter assay

HEK293T cells were co-transfected with either a GSK-3β 3′ untranslated region (3′UTR) clone or a mutant clone, and miR-129 or scramble mimics using Lipofectamine 2000 reagent. At 24 h post-transfection, the cells were assayed for luciferase activity using the Dual-Luciferase Assay System (Promega, USA) according to the manufacturer's instructions. The firefly luciferase activities were normalized to Renilla luciferase activity. The firefly luciferase activity of the cells that were transfected with miR-129 mimics was compared with cells transfected with negative control mimics. For each transfection, the luciferase activity was averaged from three replicate experiments.

### Statistical analyzes

Statistical analyzes were performed using SPSS 17.0 (SPSS, Chicago, IL, USA). Pearson correlation test was used to analyze the rank data and Mann–Whitney *U* test to differentiate the means of different groups. Kaplan–Meier survival plots were generated and comparisons were constructed with log-rank statistics. All data were shown as the mean ± standard deviation from at least three separate experiments. The differences among groups were analyzed using a double-sided Student's *t*-test, and *P* < 0.05 was considered to indicate a statistically significant difference.

## SUPPLEMENTARY MATERIALS TABLE


