# Supplementary material for: The role of glycogen synthase kinase-3β (GSK-3β) in endometrial carcinoma: A carcinogenesis, progression, prognosis, and target therapy marker
Source: Oncotarget. 2016 Mar 30;7(19):27538–51. doi: 10.18632/oncotarget.8485 (PMC5053670; doi:10.18632/oncotarget.8485)
Supplement: Supplementary file 1 [file oncotarget-07-27538-s001.pdf]

The role of glycogen synthase kinase-3β (GSK-3β) in endometrial carcinoma: A carcinogenesis, progression, prognosis, and target therapy marker

Supplementary Materials

Supplementary Table S1: Primers for RT-PCR

Gene	Primer sequence	Annealing temperature (°C)	Extension time (sec)
GSK-3β	F: 5′-GGCAGCATGAAAGTTAGCAG-3′<br>R: 5′-GGCGACCAGTTCTCCTGAAT-3′	60	34
NF-kB	F: 5′-GAAGAAGCGAGACCTGGA-3′<br>R: 5′-TCCGGAACACAATGGCCA-3′	60	34
CyclinD1	F: 5′-TGCTGGAGGTCTGCGAGGAAC-3′<br>R: 5′-AGGAAGCGTGTGAGGCGGTAG-3′	60	34
MMP9	F: 5′-GTGCTGGGCTGCTGCTTTGCTG-3′<br>R: 5′-GTCGCCCTCAAAGGTTTGGAAT-3′	60	34
P21	F: 5′-CTCAGAGGAGGCGCCATG-3′<br>R: 5′-GGGCGGATTAGGGCTTCC-3′	60	34
18s	F: 5′- ACGGACAGGATTGACAGATT -3′<br>R: 5′- GGCGTAGGGTAGGCACA -3′	60	34
